# Computed tomography imaging of septic shock. Beyond the cause: the “CT hypoperfusion complex”. A pictorial essay

**DOI:** 10.1186/s13244-021-01006-5

**Published:** 2021-06-05

**Authors:** Marco Di Serafino, Daniela Viscardi, Francesca Iacobellis, Luigi Giugliano, Luigi Barbuto, Gaspare Oliva, Roberto Ronza, Antonio Borzelli, Antonio Raucci, Filomena Pezzullo, Maria Giovanna De Cristofaro, Luigia Romano

**Affiliations:** 1 Department of General and Emergency Radiology, “Antonio Cardarelli” Hospital, Antonio Cardarelli st 9, 80131 Naples, Italy; 2 Department of Anesthesia and Resuscitation, “Antonio Cardarelli” Hospital, Naples, Italy; 3 Department of Advanced Biomedical Sciences, “Federico II” University Hospital, Naples, Italy; 4 Department of Vascular and Interventional Radiology, “Antonio Cardarelli” Hospital, Naples, Italy

**Keywords:** Sepsis, Septic shock, Diagnostic imaging, Contrast-enhanced CT, CT hypoperfusion complex

## Abstract

Septic shock is a medical emergency that represents one of the most important underlying causes for presentation to the Emergency Department. Sepsis is defined as organ dysfunction, life-threatening event caused by a deregulated inflammatory host response to infection, with a mortality risk ranging from 10 to 40%. Early sepsis identification is the cornerstone of management and diagnostic imaging can play a pivotal role in this clinical context. The choice of imaging modality depends on several factors, associated with the clinical condition and the presence or absence of localising signs and symptoms. The diagnostic accuracy of contrast-enhanced total-body CT has been well established during septic shock, allowing for a rapid, panoramic, and detailed study of multiple body areas, simultaneously. The aim of this article is to illustrate the controversial CT hypoperfusion complex in patients with septic shock, characterised by the following imaging features: decreased enhancement of the viscera; increased mucosal enhancement; luminal dilation of the small bowel; mural thickening and fluid-filled loops of the small bowel; the halo sign and flattening of the inferior vena cava; reduced aortic diameter; peripancreatic oedema; abnormal parenchymal perfusion; and other controversial findings that are variably associated with each other and reversible during the early stages. Increasing physicians’ awareness of the significance of these findings could prompt alternative approaches to the early assessment and management of septic shock. In this perspective, CT imaging represents a useful tool for a complete, rapid and detailed diagnosis of clinically suspected septic shock, which can be used to improve patient outcomes.

## Key points

- Sepsis is a life-threatening event with a mortality risk ranging from 10 to 40%.
- Early patient recognition is a cornerstone of sepsis management.
- Total-body CT imaging plays a pivotal role in septic shock condition.
- CT hypoperfusion complex recognition may facilitate early diagnosis improving treatment planning in septic shock patients.

## Introduction

Septic shock qualifies as a medical emergency and represents one of the most important causes for presentation to the Emergency Department (ED). Sepsis refers to life-threatening organ dysfunction caused by a deregulated host response to infection [1–4]. According to the new Septic 2016 definition, also referred to as Sepsis-3, septic shock patients can be identified by the co-occurrence of sepsis symptoms with persistent hypotension that requires vasopressors to maintain a mean arterial pressure of 65 mmHg or higher or serum lactate levels > 2 mmoL/L (18 mg/dL), despite adequate volume resuscitation [1, 4]. This revised and current definition emphasises that sepsis is the primary cause of death from infection [1–4]. Sepsis-associated mortality is high, ranging from 10 to 40%, and the incidence of sepsis has continuously increased in parallel with the average age of the population and the increased invasiveness of diagnostic and therapeutic procedures [4]. Mortality is associated with multiple pathogenic factors, including the timeliness of diagnosis and the execution of appropriate and early treatment [1–4]. Due to the high mortality rate, the early identification and appropriate management of sepsis are crucial to improving outcomes as well as time-dependent emergencies such as polytrauma, acute myocardial infarction, or stroke. The management of septic shock requires prompt recognition and the appropriate administration of antibiotic therapy, haemodynamic support, and the identification and treatment of the infection source [5, 6]. Early sepsis identification is the cornerstone of management, and diagnostic imaging can play a pivotal role in this clinical context. A wide range of imaging tools is currently available for the investigation of septic shock. The choice of imaging modality depends on several factors associated with the clinical condition and the presence or absence of signs and symptoms that can be used to localise the source of sepsis. The diagnostic accuracy of total-body computed tomography (CT) has been well established for the identification of septic shock, allowing for a rapid and simultaneous study of multiple body areas, generating detailed and panoramic images. The aim of this article is to review the characteristics of septic shock from an imaging perspective, beyond the underlying causes and to highlight how CT can be used to identify a variety of septic shock-related signs that are collectively described as CT hypoperfusion complex. The latter describes a set of widely reported signs and symptoms that are commonly observed during trauma-associated hypovolaemic shock and can be used to identify septic shock. The early recognition, diagnosis, and treatment of septic shock have profound prognostic and therapeutic implications.

## Etiopathogenesis

The factors that contribute to septic shock occurrence are associated with an extremely varied range of infections pathologies that can involve any area of the body. Most cases of septic shock are caused by gram-negative bacilli (*Escherichia coli*, *Klebsiella pneumoniae*, *Enterobacter* spp., *Acinetobacter baumannii,* and *Pseudomonas aeruginosa*) or nosocomial gram-positive cocci (*Staphylococcus aureus* and coagulase-negative *Staphylococcus*) cocci, and septic shock most often occurs in immunocompromised patients and in those with chronic and debilitating diseases. Antimicrobial-resistant bacteria, such as methicillin-resistant *Staphylococcus aureus* and vancomycin-resistant *Enterococci*, are often detected in septic patients with nosocomial infections. Invasive candidiasis and other uncommon pathogens should be considered in particular conditions, such as in neutropenic patients [7, 8]. The most common sites of infection include the lungs (pneumonia, empyema and lung abscess), urinary tract (obstructive urosepsis), biliary tract (cholangitis), gallbladder (acute cholecystitis), gastrointestinal tract (acute appendicitis and colic abscesses), skin/soft tissue, intravascular catheters, central nervous system, and endocardium. Subtle signs may exist, such as septic thrombosis of vascular access, mycotic aneurysms, abscess formations, and Fournier’s gangrene [3–7]. The pathogenesis of septic shock is complex, and the roles played by the inflammatory process and coagulation are highly intricate. During the initial phase, a transient dilation of the arteries and arterioles occurs, accompanied by a reduction in peripheral arterial resistance, associated with a characteristic increase in cardiac output. This pathophysiological stage has been termed “hot shock” or “high-range shock”. Subsequently, cardiac output decreases, blood pressure decreases (with or without an increase in peripheral resistance), and the typical aspects of shock appear, resulting in reduced perfusion. This cascade of responses results in the dysfunction of one or more organs, inducing disseminated intravascular coagulation (DIC) and causing death [1, 8, 9].

## Signs and symptoms

The signs and symptoms of sepsis are highly variable and clinical diagnosis often anticipates the culture results [10]. Typical signs and symptoms of sepsis include: high fever (> 38 °C), chills, diaphoresis, tachycardia, increased respiratory rate, substantial reduction of diuresis, confusion, oedema, and impairment of the general state, in addition to symptoms related to the infection. Headaches, rash, bruising, or bleeding are also common [1–4]. If left untreated, septic shock can progress to hypotension refractory to treatment, with paradoxically hot skin, oliguria, lactic acidosis, sensory alterations, and signs of impairment associated with at least one organ associated with the basic septic process. Care should be taken because sepsis can also present with nuanced manifestations that can be mistaken for other disorders, such as delirium, heart failure, and pulmonary embolism (Table 1) [1–4].

**Table 1:** Examples of diagnostic–therapeutic paths for the control and eradication of septic sources

Signs and symptoms	Laboratory tests	Diagnostic Imaging	Therapeutic strategies
Complicated urinary tract infections			
Fever, dysuria, pollakiuria, stranguria, lower back pain	Blood culture, physicochemical urine examination, urine culture	Ultrasound, contrast-enhancement US (CEUS), or CT abdomen pelvis	Antibiotic therapy; nephrostomy (if hydronephrosis); evaluation of urinary device removal
Severe pneumonia			
Fever, cough, dyspnoea	Blood cultures, respiratory secretion sampling (bronchoaspirate or bronchial lavage); urinary antigens for pneumococcus or legionella; swab for influenza virus identification (epidemic period)	Chest X-ray, thoracic ultrasound, or CT scan	Antibiotics, with or without antiviral therapy; possible drainage of pleural empyema
Complicated skin and soft tissue infections (± osteomyelitis)			
Fever, erythema, pain, oedema, suppuration, or necrosis	Blood cultures and microbiological examination of biopsy samples	Soft tissue ultrasound + bone X-ray; soft tissue + bone CT scan; soft tissue + bone MRI (magnetic Resonance imaging) (osteomyelitis*) *MRI is often the most appropriate second study, as it is highly sensitive and can detect bone marrow changes within days of infection	Antibiotic therapy; surgical therapy evaluation; hyperbaric therapy evaluation
Complicated intra-abdominal infections			
Fever, abdominal pain, signs of sepsis	Blood cultures and cultures from in situ drainage < 24 h	Abdominal ultrasound or CT scan	Antibiotic + antifungal therapy; surgical evaluation; interventional radiology evaluation
Central nervous system infections			
Fever, altered state of consciousness, signs of meningeal irritation	Blood cultures and the chemical and microbiological examination of the liquor	Head CT	Antibiotic therapy, subdural empyema drainage, or liquor derivation (hydrocephalus)
Central nervous system infections			
Fever, signs of sepsis/septic shock, local inflammation	Blood cultures and microbiological examinations of any secretions	CT scan or echocardiogram (depending on the type of infection, location, and severity)	Antibiotic + antifungal therapy; Evaluation of device removal

## Diagnosis

The diagnosis is clinical and considers various parameters, including blood pressure, heart rate and O_2_ monitoring, blood count with leukocyte formula, serum electrolytes, creatinine, lactates, the invasive monitoring of central venous pressure, PaO_2_, central venous O_2_ saturation, blood cultures, urine, and the monitoring of other potential sites of infection, especially wounds in surgical patients. Sepsis is suspected when a patient with a known infection develops systemic signs of inflammation or organ dysfunction, which requires the avoidance of septic shock and the exclusion of other potential causes for shock (e.g. hypovolemia, myocardial infarction). No gold standard diagnostic test currently exists for the identification of septic shock. However, a new bedside index, called quick Sequential Organ Failure Assessment (qSOFA) score, can be used to identify patients with suspected infection who are being treated outside of critical care units and are likely to have a prolonged intensive care unit (ICU) stay or to die in the hospital. The qSOFA requires at least 2 of following 3 risk variables: respiratory rate of 22 or more breaths per minute, systolic blood pressure of 100 mmHg or less, and altered mental status. The qSOFA does not require laboratory tests and can be assessed easily and repeatedly (Table 2) [1, 11]. Sepsis is associated with vasodilation, capillary leak, and decreased effective circulating blood volume, reducing venous return. These haemodynamic effects result in impaired tissue perfusion and organ dysfunction. Although localised signs and symptoms of organ dysfunction may be present, organ hypoperfusion or shock can manifest without knowledge of causation [10]. The goals of resuscitation in cases of sepsis and septic shock include the restoration of intravascular volume, increased oxygen delivery to tissues, and the reversal of organ dysfunction [6]. Although fluid administration will significantly increase cardiac output, the goals should be individualised for each patient, according to the evaluated need for fluids and each patient’s premorbid conditions [1, 12]. A fundamental recommendation is to initiate the necessary diagnostic process to identify the infection source while prioritising the management of haemodynamics and vital functions. The early identification of infection source and the rapid remediation of infection are essential for the appropriate management of the septic patient [1, 12].

**Table 2:** Quick sequential organ failure assessment (qSOFA) score

Quick sequential organ failure assessment (qSOFA)		Score
Respiratory rate ≥ 22/min		1
Change in mental status		1
Systolic blood pressure ≤ 100 mmHg		1

## Diagnostic imaging

The importance of imaging for the establishment of the infection source is well recognised [1]. A wide range of imaging tools is currently available for the investigation of septic shock. The choice of imaging modality depends on several factors, the most important of which include the overall clinical condition of the patient and the presence or absence of localising signs and symptoms as summarised in Table 1 [12–21].

The diagnostic accuracy of CT for the identification of the source of clinically suspected septic shock has been well established, allowing for the rapid and detailed study of multiple body areas simultaneously. CT provides a standardised method for patient evaluation, which is not operator-dependent. In a few minutes, CT can provide images of multiple body regions, simultaneously, improving the ability to identify the septic source, which can improve patient management [1, 22]. CT also played an increasingly important role in the guidance of aspiration and drainage, with a high degree of accuracy [16]. CT should be performed in all patients with a spiral technique, craniocaudal acquisition and in a supine position with abducted upper limbs, to reduce the radiation dose and guarantee a higher image quality of the thoraco-abdominal organs. Breath-hold acquisitions can ensure the avoidance of motion artefacts [22]. In emergency settings, CT examinations should include both unenhanced and contrast-enhanced acquisitions of the abdomen. High concentrations (370–400 mg I/mL) of intravenous (IV) contrast medium (80–130 mL iodinated contrast medium, depending on the patient’s weight), injected at 3.5–4 mL/s through an 18-gauge needle into the antecubital vein should be administered, followed by a bolus of 40 mL saline at the same flow rate. The acquisition of the arterial phase is timed with the bolus tracking by placing the region of interest (ROI) on the aortic arch and starting at an attenuation threshold of 100 Hounsfield Unit (HU). The portal-venous phase is acquired with a delay of 60–70 s after the beginning of the injection. The suggested acquisition volume in emergency settings includes the abdomen and pelvis scan in arterial phase and the chest, abdomen, and pelvis in portal phase, to obtain a complete examination. An additional, late scan of the abdomen and pelvis at 3–5 min may also be acquired to address various causes of abdominal pain. The rectal administration of contrast material is not typically useful. For adequate analysis and post-processing, with maximum intensity projection (MIP) and multiplanar reformation (MPR), an effective slice thickness of 2.5 mm, with reconstruction at 0.625 mm, is recommended. Automatic tube current modulation should be adopted to reduce radiation exposure, and the standard reconstruction algorithm should be applied. Head CT should be considered for patients presenting with altered mental status, without IV contrast medium administration; head CT should be performed before the total body study to exclude the presence of intracranial haemorrhage. Head CT can also be performed after the late phase of the total body study to exclude intracranial abscess or malignancy [22].

## CT hypoperfusion complex

In addition to being used to identify the underlying cause of the septic state, CT is required for the early recognition of shock-associated CT imaging signs, collectively referred to as CT hypoperfusion complex, which can improve patient prognosis and management. The CT hypoperfusion complex is frequently associated with hypotension, which can also present in many no sepsis related clinical conditions, such as trauma-induced hypotensive shock (e.g. severe head or spine injury), cardiac arrest, and diabetic ketoacidosis [23–26]. The CT hypoperfusion complex has important prognostic and therapeutic implications and must be promptly recognised. However, although the pathogenic mechanisms that underlie hypotensive shock and septic shock are quite different, the CT findings associated with these two syndromes are often comparable to those that have been widely described in previous literature as in post-traumatic hypotensive shock, which can be grouped into vascular, visceral, and parenchymal signs. These signs include the decreased enhancement of the viscera, the increased mucosal enhancement and luminal dilation of the small bowel, the mural thickening and identification of fluid-filled loops in the small bowel, the halo sign and flattening of the inferior vena cava (IVC), reduced aortic diameter, peripancreatic oedema and other controversial parenchymal and visceral findings and ascites that can occur in varying combinations and are often and reversible during early stages. The presence of 2 or more vascular, visceral, or parenchymal signs is necessary to establish the presence of CT hypoperfusion complex [20–23] (Table 3).

**Table 3:** CT hypotension complex findings and frequency

CT hypoperfusion complex			
Type	Sign	Definition	Incidence rate in patient with severe hypoperfusion*
Vascular signs	Flattening of the inferior vena cava	IVC flattening with anterior-posterior diameter < 9 mm in three consecutive segments, 20 mm above and below the renal veins and at the level of the perihepatic portion	77–100%
	The halo sign	The presence of low-density fluid < 20 HU surrounding the IVC	77.8%
	Small calibre aorta	A small-calibre abdominal aorta with anterior–posterior diameter < 13 mm detected 20 mm above and below the renal arteries	20–48%
Visceral sign	Shock bowel	Small bowel fluid-filled dilated loops with thickened walls (> 3 mm) due to oedema of the submucosa and increased mucosal enhancement relative to the psoas muscle	40–70%
	Mucosal enhancement of the gallbladder	Mucosal enhancement, without thickened gallbladder walls	33.3%
Parenchymal signs	Peripancreatic oedema and abnormal pancreatic enhancement (shock pancreas)	Abnormal post-contrast higher attenuation than to normal density values (20 HU greater than the liver and spleen) often with peripancreatic low-density fluid (< 20 HU)	> 44%
	Splenic hypoperfusion	Extremely decreased enhancement in contrast early CT phase	11–29.6%
	Abnormal liver enhancement	A reduction of hepatic enhancement (25 HU less than the spleen)	4–11.1%
	Abnormal renal enhancement	Increased and prolonged parenchymal enhancement	55.6%
	Abnormal adrenal hyperenhancement (adrenal stress)	Bilateral hyperenhancement of the adrenal gland	> 60%
	Abnormal thyroid enhancement (shock thyroid)	Heterogeneous contrast hyperenhancement, similar to a multinodular gland, with the presence of low-density fluid surrounding the thyroid (5–10 HU)	Not detected
Other sign	Ascites	Fluid collects in peritoneal spaces	Not detected

*Literature data from post-traumatic hypoperfusion complex [26]

Vascular signs include diminished inferior vena cava diameter, diminished aortic diameter, and abnormal vascular enhancement.

### Flattening of the inferior vena cava

**Fig. 1:**
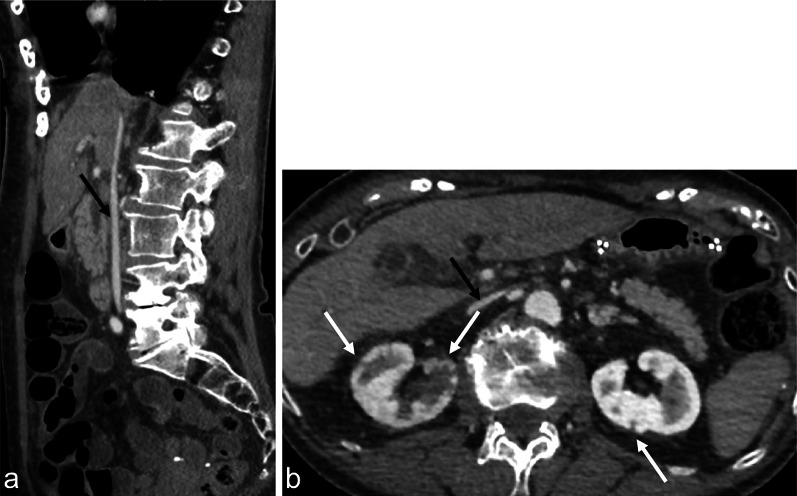
Contrast-enhanced CT in the portal venous phase, showing an 84-year-old male with sepsis (qSOFA 2) due to bilateral nephritis (white arrows). A collapsed IVC can be observed in both the sagittal (**a**, black arrow) and axial views (**b**, black arrow)

The flattening of the IVC calibre has been defined as the identification of reduced anterior–posterior diameter (< 9 mm) in three consecutive segments, 20 mm above and below the renal veins, and at the level of the perihepatic portion (Fig. 1). Flattening is the result of decreased circulating blood volume and indicates reduced venous return in patients with systemic hypotension, which may not be appreciable due to the massive infusion of liquids. In addition, variations in intra-abdominal pressure and the respiratory cycle can also affect the IVC diameter. IVC flattening has a specificity of 90% and a sensitivity of 84% for the identification of hypoperfusion shock due to sepsis in spontaneously breathing patients, whereas the sensitivity and specificity are both 90% in ventilated patients [20–23].

### The halo sign

**Fig. 2:**
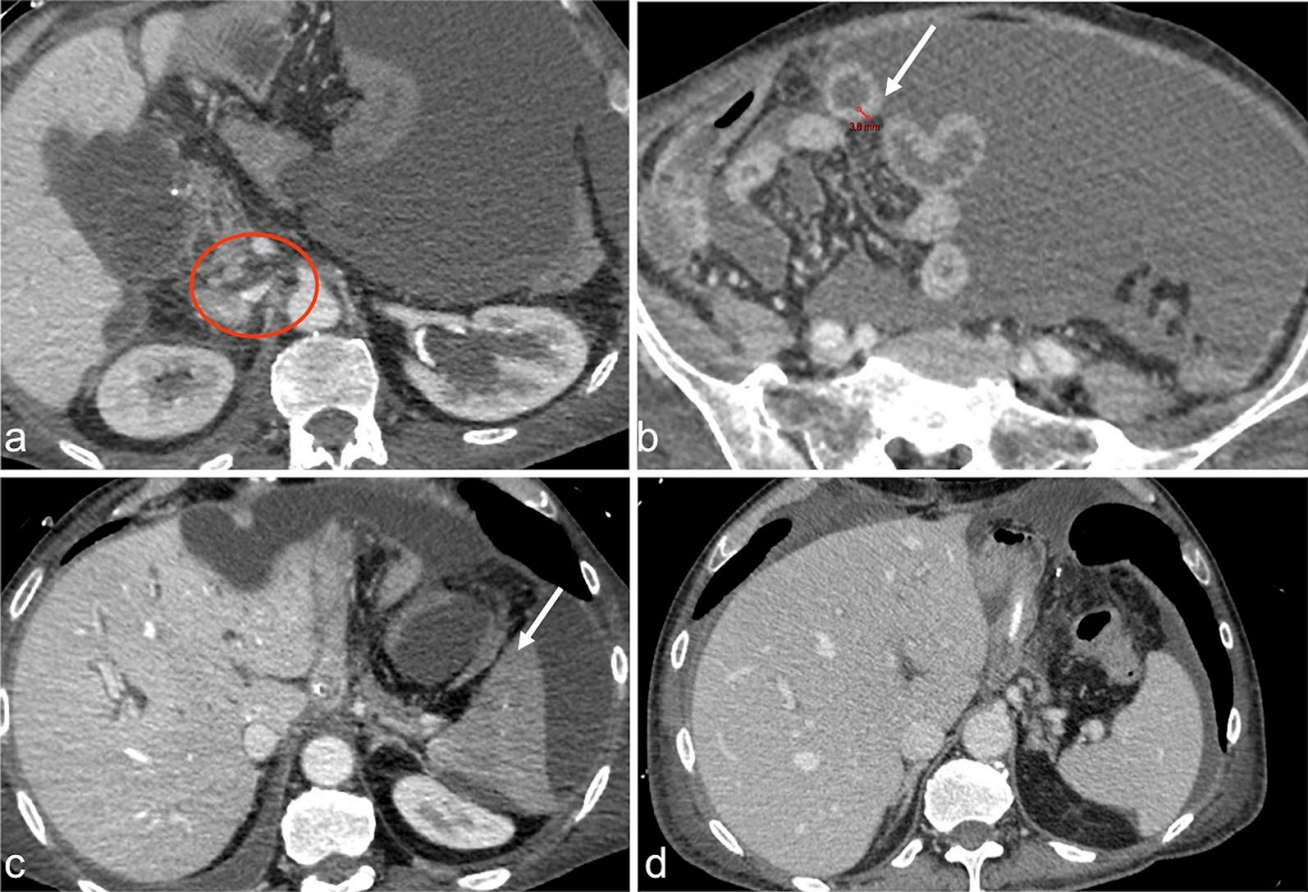
Contrast-enhanced CT in the portal venous phase, in the axial view, showing a 70-year-old male with sepsis due to gastric cancer surgery complications (qSOFA 3). In this patient, multiple CT signs of sepsis can be observed, including inferior vena cava halo sign (**a**, red circle), thickened bowel wall (**b**, white arrow), reduced enhancement, and spleen volume (**c**, white arrow). Figure d shows a previous CT examination of the same patient for comparison

The presence of low-density fluid (< 20 HU) surrounding the IVC, known as an IVC halo, can be identified in approximately 80% of patients with severe hypotension (Fig. 2a). The IVC halo represents the presence of extracellular fluid, which may be due to a hyperpermeable state, secondary to systemic inflammatory response syndrome [23]. The halo sign is most frequently observed at the confluence of the hepatic veins into the IVC. The halo sign is not specific to hypoperfusive shock due to sepsis and can also be observed in other clinical settings, such as liver cirrhosis, congestion, or hepatitis [20–23].

### Small-calibre aorta

**Fig. 3:**
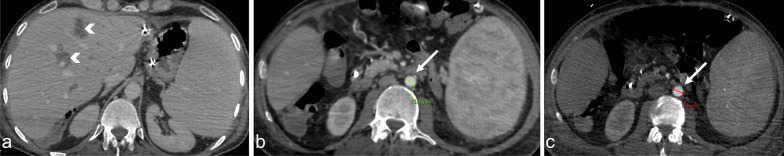
Contrast-enhanced CT in the portal venous phase, in the axial plane, showing a 44-year-old female with sepsis (qSOFA 2) due to cholangitis with liver abscesses (**a**, arrowheads). The CT revealed the reduced calliper of the abdominal aorta (**b**, arrow), compared with the normal calliper of the abdominal aorta in a previous examination from the same patient (**c**)

A small-calibre abdominal aorta is defined as a reduced anteroposterior diameter (< 13 mm) detected 20 mm above and below the renal arteries (Fig. 3). Small-calibre aorta occurs in approximately 30% of patients with systemic hypotension and is not specific to hypoperfusive shock due to sepsis, as it may be observed in the normal population. This sign is associated with vasoconstriction induced by the adrenergic system to compensate for the shock condition [23–27].

*Visceral signs* include bowel hypoperfusion also called shock bowel and mucosal enhancement of the gallbladder.

### Bowel hypoperfusion (shock bowel)

**Fig. 4:**
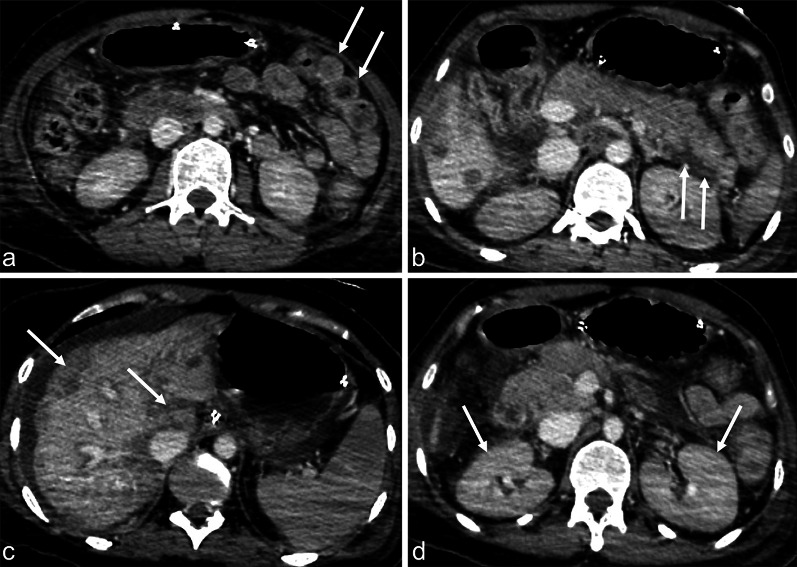
Contrast-enhanced CT images in the portal venous phase, in the axial view, showing a 46-year-old female with lymphoma and sepsis due to infection (qSOFA 2). A thickened and hyperdense bowel wall can be observed (**a**, arrows), with peripancreatic oedema (**b**, arrows), reduced and inhomogeneous liver enhancement (**c**), and enlarged kidneys with abnormal enhancement (**d**, arrows)

The most frequent findings associated with shock bowel include fluid-filled, dilated loops with thickened walls (> 3 mm) due to oedema of the submucosa and increased mucosal enhancement relative to the psoas muscle (Figs. 2b, 4a). Changes to the small intestine are the most commonly observed characteristics among the CT signs indicative of shock. The small intestine is often diffusely involved in the occurrence of hypotensive shock, whereas the colon is rarely involved [23–26]. The shock bowel symptoms occur due to systemic hypotension, with consequent sympathetic stimulation, resulting in splanchnic vasoconstriction and a reduction in intestinal perfusion. These effects reduce the supply of oxygen to the tissues, altering permeability and causing the hyperenhancement of the mucosa and the oedematous thickening of the intestinal wall and submucosa. The reduced reabsorption of fluids causes the luminal distension of the intestinal loops. The recognition of the shock intestine is essential to avoid confusion with other conditions, such as intestinal ischaemia due to vascular occlusion, which are associated with different CT characteristics. The most challenging differential diagnosis is diffuse bowel ischaemia due to vascular occlusion, which can also present with bowel-wall thickening and luminal distension. Unlike the shock bowel, bowel ischaemia due to arterial occlusion is not associated with the hyperenhancement of the mucosa or the congestion of the wall. Mesenteric venous occlusion may show both of these CT signs, however, in combination with a filling defect of the superior mesenteric vein or its branches, mesenteric congestion, and stranding [23]. In addition, CT signs associated with hypotension complex can be important indicators of systemic hypotension, which may facilitate the differential diagnosis. Shock bowel has a mortality rate of up to 70% [23–26, 28–31].

### Mucosal enhancement of the gallbladder

**Fig. 5:**
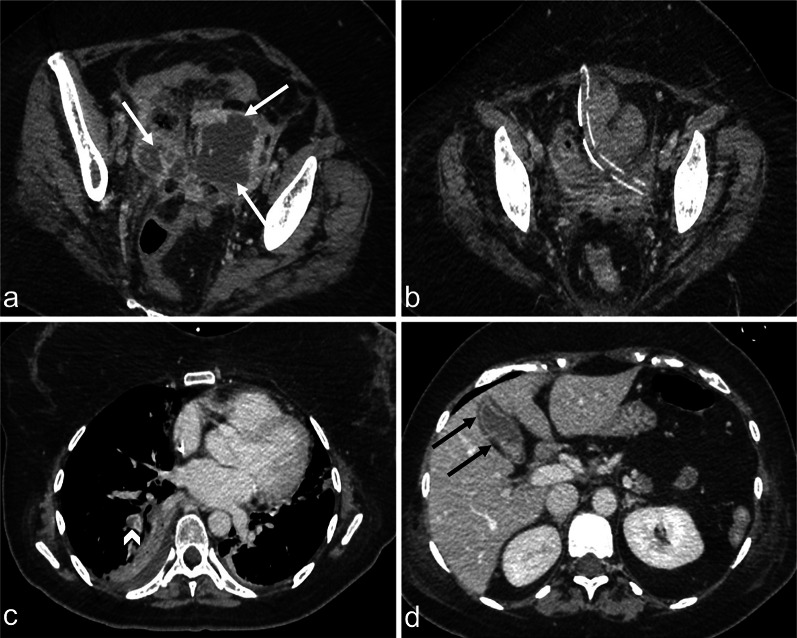
Contrast-enhanced CT image in the portal venous phase showing a 44-year-old female with abscessed uterine neoplasm (**a**, arrows) and post-operative control with completely drained collection (**b**). Pulmonary thromboembolism (**c**, arrowhead) with development of a clinical septic state (qSOFA 2). Note the dense, gallbladder mural enhancement without thickened walls (**d**, black arrows)

Mucosal enhancement, without thickened gallbladder walls, can be observed in hypotensive shock complex, with low specificity (Fig. 5) [23].

*Parenchymal signs* include abnormal pancreatic enhancement, associated with peripancreatic fluid, the hypoperfusion of the spleen and liver and the abnormal perfusion of the adrenal glands, kidneys and thyroid.

### Peripancreatic oedema and abnormal pancreatic enhancement (shock pancreas)

**Fig. 6:**
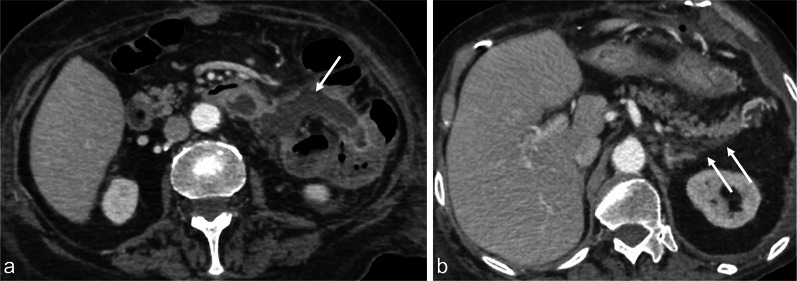
Contrast-enhanced CT images in the portal venous phase, in the axial view, shows a 78-year-old female with sepsis (qSOFA 2) due to bowel anastomotic dehiscence (**a**, the white arrow indicates fluid collection caused by an anastomotic leak). Peripancreatic oedema can also be observed (**b**, white arrows)

Shock pancreas appears as an abnormal, post-contrast attenuation, with higher than normal density values (20 HU greater than the liver and spleen) and the presence of peripancreatic low-density fluid (< 20 HU), often in combination with mesenteric and other retroperitoneal fluid collections, at an incidence rate of up to 44% (Figs. 4b and 6). This phenomenon may be the result of cytokine release, due to systemic inflammatory response syndrome and pancreatic ischaemia, causing increased capillary permeability and the loss of intravascular oncotic pressure; however, this sign can also be identified in patients with pancreatitis [23–27, 32, 33].

### Splenic hypoperfusion

The spleen is among the most vulnerable abdominal organs to hypotensive shock and often shows extremely decreased enhancement in early-phase CT (Figs. 2c–d). The hypoenhancement of the spleen and a reduction in the splenic volume are associated with severe hypoperfusion. The degree of hypoenhancement is believed to be directly related to hypoperfusion due to shock because the splenic artery has not autoregulatory mechanisms. Splenic hypoperfusion appears to be a useful predictor of poor prognosis among patients with systemic hypotension [23–27, 31].

### Liver hypoperfusion

Hepatic enhancement is typically heterogeneous during hypotensive shock complex. A reduction in hepatic enhancement (25 HU less than the spleen) is thought to be significant due to oedema (Fig. 4c) [23]. This CT manifestation is less common than other solid parenchymal abnormalities. False-positive interpretations could occur in patients with diffuse underlying liver disease, such as hepatic steatosis or liver congestion venous stasis [23–27]

### Abnormal renal enhancement

**Fig. 7:**
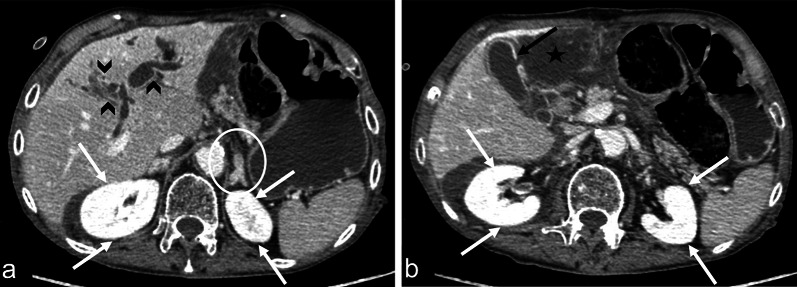
Contrast-enhanced CT images in the portal venous phase, in the axial view, shows a 92-year-old male with sepsis of the biliary tract (qSOFA 3) characterised by segmental intrahepatic biliary duct dilatation (**a**, black arrowheads) and gallbladder leak (**b**, black arrow) with extrahepatic biloma (**b**, black star). Note the increased renal parenchymal enhancement bilaterally (**a**, **b**, white arrows) and the abnormal adrenal enhancement (**a**, white circle)

**Fig. 8:**
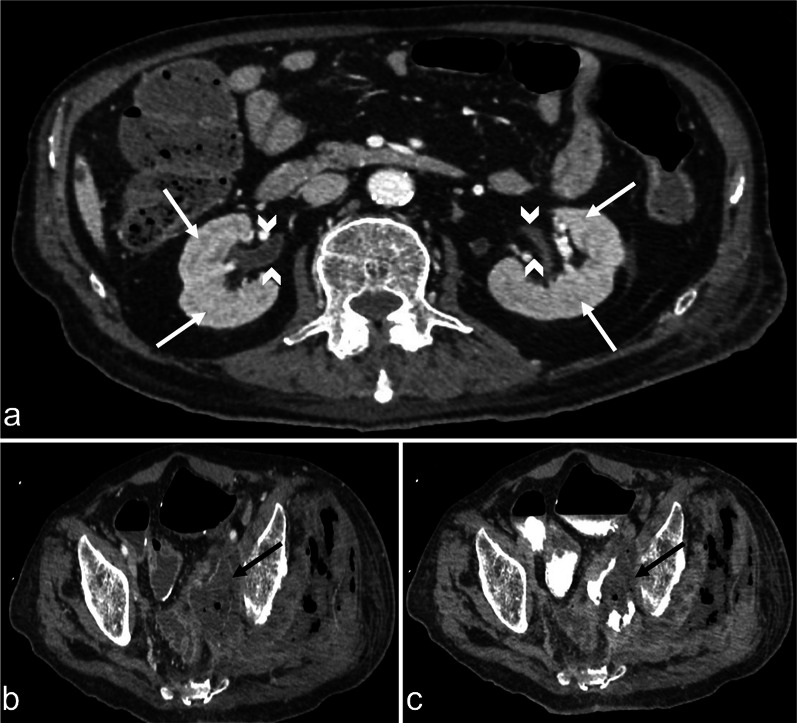
Contrast-enhanced CT images in the portal venous phase (**a** and **b**) and the three-minute-delayed phase (**c**), showing a patient with septic shock (qSOFA 2) due to entero-neovesical fistula (**b** and **c**, black arrows) with pyelitis (**a**, arrowheads) after radical cystectomy. The decreased enhancement of the renal medulla (**a**, white arrows) in the venous phase was observed

Abnormal renal perfusion typically manifests as an increased and prolonged parenchymal enhancement (Fig. 7); however, focal and heterogeneous enhancement can also be observed. A fall in systolic pressure causes intense efferent glomerular arteriolar vasoconstriction, which drives glomerular filtration, leading to tubular stasis and the increased resorption of salt and water. Renal parenchymal enhancement is dependent on several factors, including cardiac output and scans timing relative to the injection of contrast agent and, thus, is a non-specific sign [23–27, 31]. However, kidney enhancement can vary depending on the severity of systemic hypotension. In some cases, unlike hyperenhancement, the decreased enhancement of the renal medulla can be observed in the venous phase, likely due to the impairment of contrast medium outflow from the renal cortex to the medulla, induced by acute renal tubular dysfunction and associated with poor prognosis (Figs. 4d, 8) [26].

### Abnormal adrenal enhancement (the adrenal stress)

**Fig. 9:**
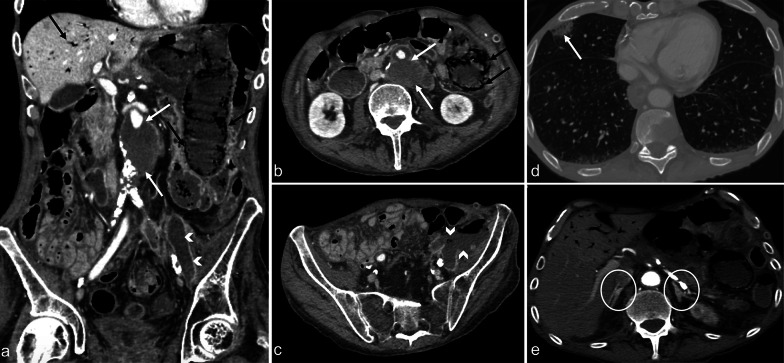
Contrast-enhanced CT images in the arterial phase showing a 75-year-old male with sepsis (qSOFA 3) due to an infected aneurysm after aorto-basilic stent placement (**a** and **b**, white arrows). Concomitant intestinal images reveal liver septic pneumatosis (**a** and **b**, black arrows), stercoraceous collection in the left iliac fossa (**c**, white arrowheads), and septic emboli with pulmonary infarction (**d**, white arrows). The adrenal glands display hyperenhancement (**e**, white circles)

**Fig. 10:**
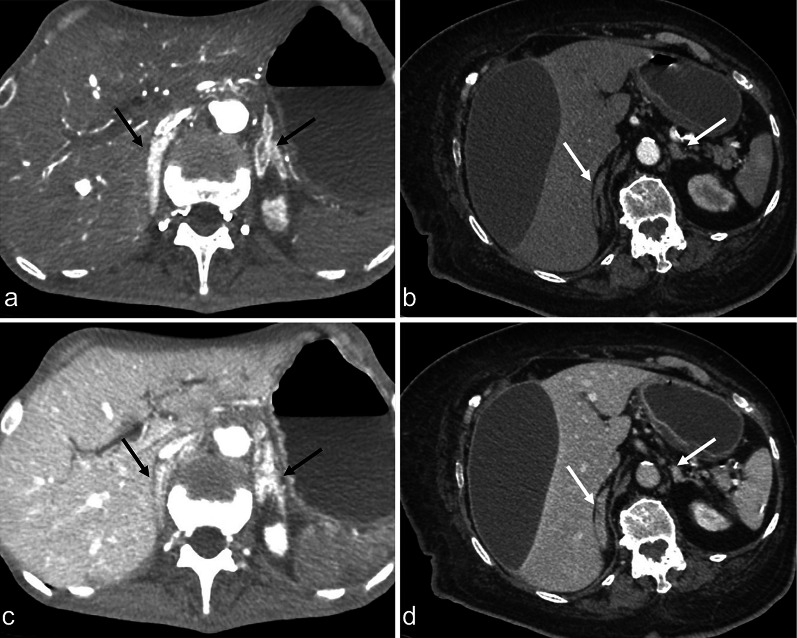
Contrast-enhanced CT images in the arterial phase from two different patients with septic shock (**a** and **b**) and the respective venous phases (**c** and **d**). Different patterns of adrenal gland hyperenhancement can be observed, characterised in one patient by a central zone with less intense enhancement relative to the peripheral zone (**a**, black arrows) and in the other patient by a central zone with heterogeneous enhancement and a mosaic-like appearance (**b**, white arrows). In both cases, the venous phase shows a homogeneous enhancement (**c**, black arrows; **d**, white arrows)

The bilateral hyperenhancement of the adrenal gland is more common in paediatric cases than in adults and can also present in combination with acute adrenal haemorrhage, which most commonly affects the right side unilaterally, with a homogeneous increase in the size of the gland and the associated suffusion of fat around the adrenal gland (Figs. 7a, 9). Bilateral adrenal hyperenhancement is the manifestation of adrenergic mechanisms that enhance the blood flow to the vital organs [23–27, 34, 35]. In the arterial phase, the central zone of the adrenal gland shows less intense enhancement than the peripheral zone or presents a mosaic appearance due to the heterogeneous enhancement of the central zone (Fig. 10). In both cases, in the venous phase, the whole adrenal gland is homogenously enhanced [36]. This sign highlights the central role played by the adrenal glands in mediating the sympathetic response to hypotensive shock and is associated with poor prognosis [23–27, 34, 35].

### Abnormal thyroid enhancement (shock thyroid)

**Fig. 11:**
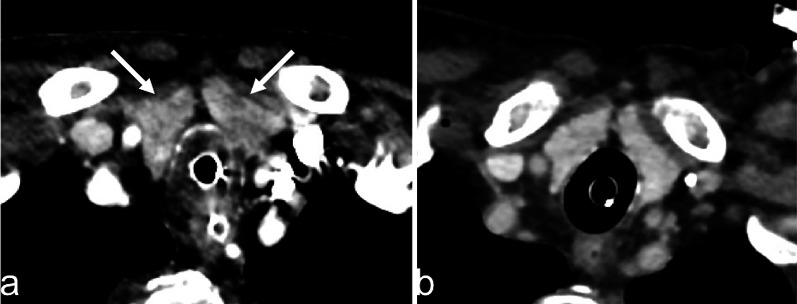
Contrast-enhanced CT image in the portal venous phase showing an 89-year-old female with sepsis due to bowel wall perforation, secondary to colon cancer (qSOFA 3). An inhomogeneous enhancement of the thyroid gland was observed (**a**, arrow), in contrast with the previously homogeneous enhancement of the gland 4 days earlier (**b**)

The thyroid gland, in the absence of direct damage, presents an increase sized, heterogeneous contrast hyperenhancement, similar to a multinodular gland, with the presence of low-density fluid surrounding the thyroid (5–10 HU) (Fig. 11). Thyroid shock is a minor finding associated with hypotensive shock complex in the absence of any known direct or indirect thyroid injury [23–27, 37, 38].

## Other sign

### Ascites

The presence of ascites correlates with multiorgan dysfunction and is a non-specific sign [39].

## Conclusion

In previously published studies, CT hypoperfusion complex has been almost exclusively focused on trauma-induced hypotensive shock and only few studies correlated these signs with prognosis. Some studies have suggested that flattering of IVC, shock bowel, impaired renal enhancement and splenic hypoperfusion are the most suggestive signs of hypoperfusion complex, strongly correlated with a poor prognosis although in a series of trauma-related hypovolemic shock [23–26, 31]. In contrast, only adrenal hyperenhancement has been correlated with a poor prognosis in septic shock [36]. Despite these contradictory reports, understanding these findings could prompt the development of alternative approaches for the early assessment and management of septic shock in the emergency setting [26]. Therefore, in addition to the application of CT for determining the underlying cause of the septic state, clinicians should be aware and be able to recognise the various CT findings that are suggestive of the hypotensive state. In this perspective, CT imaging represents a useful tool for a complete, rapid, and detailed diagnosis of clinically suspected septic shock, which can be used to improve patient outcomes.

## Data Availability

Data sharing is not applicable to this article as no datasets were generated or analysed during the current study.

## Abbreviations

ARDS: Acute respiratory distress syndrome
CEUS: Contrast-enhancement ultrasound
CT: Computed tomography
DIC: Disseminated intravascular coagulation
ED: Emergency department
HU: Hounsfield unit
ICU: Intensive care unit
IV: Intravenous
IVC: Inferior vein cava
MAP: Mean arterial pressure
MIP: Maximum intensity projection
MPR: Multiplanar reformation
MRI: Magnetic resonance imaging
qSOFA: Quick sequential organ failure assessment
ROI: Region of interest
US: Ultrasound
